# Differentiation of lipoma and atypical lipomatous tumor by a scoring system: implication of increased vascularity on pathogenesis of liposarcoma

**DOI:** 10.1186/s12891-015-0491-8

**Published:** 2015-02-22

**Authors:** Satoshi Nagano, Masahiro Yokouchi, Takao Setoguchi, Yasuhiro Ishidou, Hiromi Sasaki, Hirofumi Shimada, Setsuro Komiya

**Affiliations:** Department of Orthopaedic Surgery, Graduate School of Medical and Dental Sciences, Kagoshima University, 8-35-1 Sakuragaoka, Kagoshima-city, Kagoshima 890-8520 Japan; The Near-Future Locomotor Organ Medicine Creation Course (Kusunoki Kai), Graduate School of Medical and Dental Sciences, Kagoshima University, Kagoshima, Japan; Department of Medical Joint Materials, Graduate School of Medical and Dental Sciences, Kagoshima University, Kagoshima, Japan

**Keywords:** Atypical lipomatous tumor, Magnetic resonance imaging, Scoring system, Tumor angiogenesis, Dedifferentiation

## Abstract

**Background:**

Well-differentiated liposarcoma (WDL)/atypical lipomatous tumor (ALT) is considered a low-grade malignancy that rarely metastasizes but should be carefully followed because recurrence or dedifferentiation may occur. It is recognized that WDL and ALT are essentially synonymous, describing lesions that are identical both morphologically and karyotypically, and that site-specific variations in behavior relate only to surgical resectability. Preoperative differential diagnosis between lipoma and ALT has been well studied because their clinical and image characteristics are very similar. We evaluated the factors that may differentiate ALTs from lipomas, and validated a tentative scoring system for the diagnosis of the 2 tumor types.

**Methods:**

Forty-eight lipomas and 12 ALTs were included. The mean age, location and depth of the tumor as well as the compartment were not significantly different between the 2 groups. To evaluate the vascularity of the tumors, the average number of intratumoral vessels on pathological sections was calculated and compared between cases of lipoma and ALT.

**Results:**

The tumor size was significantly larger in ALT cases than in lipoma cases (*P* < 0.001). Magnetic resonance imaging (MRI) revealed septal structures in 91.6% of ALTs, whereas 20.8% of lipomas showed septa. Contrast enhancement in MRI was found significantly more often in ALTs (81.2%) than in lipomas (18.8%) (*P* < 0.001). We created a “ALT score” to discriminate between lipoma and ALT (0–6 points). ALT cases gave significantly higher point values (average 5.1 points) than lipoma cases (average 1.7 points) (*P* < 0.001). We found a significantly increased number of vessels in cases of ALT than in cases of lipoma (*P* = 0.001).

**Conclusions:**

Our ALT score may help surgeons to differentiate a suspected ALT from a lipoma and could recommend a marginal resection in cases of suspected ALT. Increased intratumoral vascularity in ALT is reflected in the MRI findings and may play a key role in the acquisition of a malignant phenotype in adipocytic tumors.

## Background

Adipocytic tumors are the soft tumors most frequently encountered by orthopaedic physicians in clinics. Benign adipocytic tumors, lipomas, can be conservatively observed unless patients experience symptoms due to the presence of the mass. However, tumors that are preoperatively suspected to be lipomas, can sometimes be intermediate (locally aggressive)-type adipocytic tumors or well-differentiated liposarcoma (WDL)/atypical lipomatous tumors (ALTs). WDL is considered a low-grade malignancy that rarely metastasizes but should be carefully followed because recurrence or dedifferentiation may occur [1]. It is recognized that WDL and ALT are essentially synonymous, describing lesions that are identical both morphologically and karyotypically, and that site-specific variations in behavior relate only to surgical resectability [2]. The term WDL is now used for tumors of the retroperitoneum, mediastinum, and deep pelvis, whereas the term ALT includes tumors of the extremities and superficial sites. Preoperative differential diagnosis between lipoma and WDL/ALT has been well studied because their clinical and image characteristics are very similar [3,4]. Magnetic resonance imaging (MRI) is currently the most popular modality for the screening and diagnosis of soft tissue tumors, including adipocytic tumors. MRI findings of lipomas usually show high intensity in both T1- and T2-weighted images, reflecting their uniform structure with fatty tissue. In contrast, high-grade liposarcomas, including myxoid, round cell, pleomorphic, and dedifferentiated liposacrcoma, show low intensity in T1-weighted images. The MRI features of WDL/ALT are similar to those of lipoma, which makes differentiation between them difficult. In general, a larger size, deeper localization, or enhancement with contrast medium in MRI is suggestive of malignant soft tissue tumors. In this study, we evaluated the factors that may differentiate ALTs from lipomas and aimed to establish a feasible scoring system to help in the diagnosis of the 2 tumor types. Furthermore, we examined if increased vascularity in the surgical specimen could be a finding that pathologically differentiates ALTs from lipomas, and affect the clinical behavior of ALT.

## Methods

We retrospectively reviewed the records of 48 patients with lipomas and 12 patients with ALT. According to the definitions of WDL and ALT, tumors of the extremities and superficial trunk come under the term ALT [2]. In this series, we aimed to study tumors of the extremities and superficial trunk treated in our department of orthopedic surgery. Therefore, no cases of WDL were included in this study. All patients underwent surgical excision of the tumor, and a pathologist established the pathological diagnosis. Age, sex, tumor location (limb or trunk), size (diameter in MRI), and depth (superficial, subcutaneous or deep, or under the fascia), and intracompartmental or extracompartmental location were evaluated in all cases. In the MRI analysis, the presence of septal structures (more than 2 mm thick) was assessed. On fat-suppressed T1-weighted images after the administration of contrast-enhancing medium, enhancement of intratumoral lesions was evaluated in all cases. All tumors were resected by marginal resection, and pathological diagnosis was established by pathologists.

To evaluate the vascularity of the tumors, the number of vessels was counted in 10 randomly taken microscopic pictures of hematoxylin and eosin stained sections. The average number of intratumoral vessels was calculated and compared between cases of lipoma and ALT.

The average value of age and tumor size was analyzed with a Student’s *t*-test. All other factors were analyzed using the Chi-square test. A *P* value of less than 0.05 was considered significant.

The ethical committee in Kagoshima University approved the study (reference number, 353).

## Results

**Figure 1:**
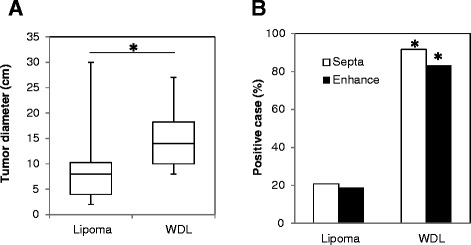
**Comparison of magnetic resonance imaging (MRI) findings in cases of lipoma and well-differentiated lipoma (ALT). (A)** The tumor diameter was significantly larger in ALTs than lipomas (*P < 0.001). **(B)** Septa formation and contrast enhancement were found in most of the ALT cases and rarely in lipoma cases (*P < 0.001).

The mean ages of the patients with lipoma and ALT were 59 (range, 27–77) and 62 (range, 44–78) years, respectively, and the difference was not statistically significant (*P* = 0.22). The location (trunk or extremity) and depth of the tumor (superficial or deep) and intracompartmental or extracompartmental location were not significantly different between the 2 groups (Table 1). The tumor size was significantly larger in cases of ALTs (average, 15.3 cm) than in cases of lipomas (average, 8.9 cm) (*P* < 0.001, Figure 1A). On T2-weighted MRI, septal structures were found in 11 of 12 (91.6%) cases of ALT, whereas 10 of 48 (20.8%) cases of lipoma showed septa (*P* < 0.001, Figure 1B). ALT was significantly intensively enhanced by gadolinium in MRI in 81.2% ALT cases, whereas the incidence was 18.8% in lipoma cases (*P* < 0.001, Figure 1B).

**Table 1:** **Summary of patient characteristics**

	**Lipoma**	**ALT**	**P-value**
	Average (range)		
**Age**	59 (27–77)	62 (44–80)	0.27
**Gender**	Cases (%)		
**Male**	25 (52.1)	7 (58.3)	0.70
**Female**	23 (47.9)	5 (41.7)	
**Location**			
**Extremity**	29 (60.4)	8 (66.7)	0.70
**Trunk**	19 (39.6)	4 (33.3)	
**Depth**			
**Superficial**	24 (50.0)	4 (33.3)	0.30
**Deep**	24 (50.0)	8 (66.7)	
**Compartment**			
**Intracompartment**	5 (16.1)	2 (16.7)	0.97
**Extracompartment**	26 (83.9)	10 (83.7)

**Figure 2:**
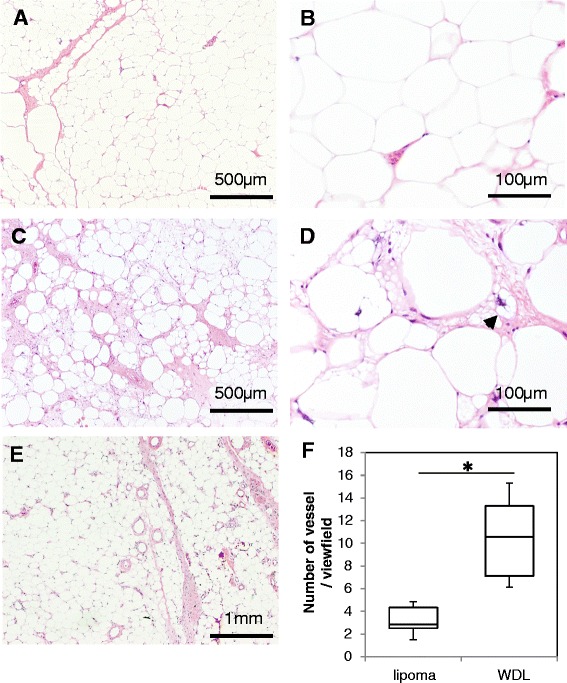
**Pathological analysis and evaluation of intratumoral vascularity.** In lipoma specimens, mature adipocytes were uniformly observed without high variation in size **(A, B)**. By contrast, adipocytes in ALT showed significant variation in size **(C)**, and lipoblasts with cytoplasmic vacuoles were occasionally found around the thick septa **(D)**. Septa in ALTs were thicker than those in lipomas, and vessel formation was observed both inside and outside of the thick septa **(E)**. Vascular formation was found significantly more often in ALTs than in lipomas **(F)** (*P = 0.001). Original magnification, ×20 **(E)**, ×40 **(A, C)**, ×100 **(B, D)**.

In pathological examination of the lipoma specimens, mature adipocytes were uniformly arranged without high variations in size (Figure 2A,B). In contrast, adipocytes in ALT showed marked variation in size, and many hyperchromatic stromal cells were found around the thick septa (Figure 2C,D). Monovacuolated or multivacuolated lipoblasts are considered a hallmark of liposarcoma, although WDL/ALTs do not always contain lipoblasts. In our study, atypical lipoblasts with cytoplasmic vacuoles and scalloped nuclei were seen in some cases (Figure 2D). Although vessels were found inside the fibrous septa in both lipomas and ALTs, septa were much less frequently found in lipomas. In addition, septa in ALTs were thicker than those in lipomas, and vessel formation was observed both inside and outside of the thick septa (Figure 2E). Analysis of the vascularity in the tumors revealed that ALTs had significantly more vessels (average 11.1/view field) than lipomas (average 3.82/view field) (*P* = 0.001, Figure 2F).

**Figure 3:**
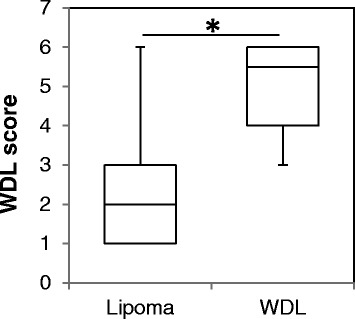
**Comparison of the ALT score (0–6 points) in lipoma and ALT groups.** The majority of lipoma cases showed a ALT score of less than 2 points, whereas the ALT cases showed significantly high points values (average of 5.1 points) (*P < 0.001).

In order to develop a new diagnostic tool for adipocytic tumors, we created a scoring system to discriminate between lipomas and ALTs by considering the tumor size, depth, septa, and enhancement on MRI (Table 2). Total points for the scoring system ranged from 0 to 6 depending on the positivity of those findings, and we expected that a high number of points would suggest the increased probability of the diagnosis of ALT. Almost all lipoma cases gave low scores (average 1.7 points), whereas ALT cases gave significantly high point values (average of 5.1 points) (*P* < 0.001, Figure 3). Based on this scoring system, the diagnosis of ALT was possible with 100% sensitivity and 77% specificity.

**Table 2:** **Scoring for the diagnosis of ALT**

**Value**	**Points**
**Diameter (cm)**	
<10	0
≥10	1
**Depth**	
Superficial	0
Deep	1
**Septa (MRI)**	
No	0
Yes	2
**Enhancement (MRI)**	
No	0
Yes	2

One of the 12 ALT patients had a recurrence with dedifferentiation 4 years after resection. At the time of dedifferentiation, the entire tumor measured 28 cm and contained 5 cm of a dedifferentiated lesion. The tumor was deep-seated, had septa, and was enhanced by gadolinium in MRI (6 points by the ALT scoring system). This patient underwent wide resection of the ALT and dedifferentiated lesion. Although there was no sign of recurrence or metastasis 3 months after the resection, careful follow-up is required.

## Discussion

**Figure 4:**
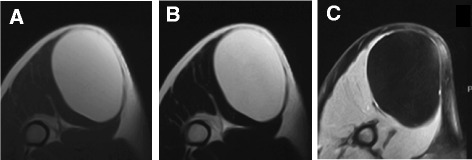
**A 34-year-old male patient with a lipoma measuring 12 cm in diameter.** Axial T1- and T2- weighted MRIs showed a homogenously high intramuscular mass **(A, B)**. T1-weighted fat saturation gadolinium-enhanced MRI showed no enhancement of the tumor **(C)**. This case obtained 2 points using the ALT score.

**Figure 5:**
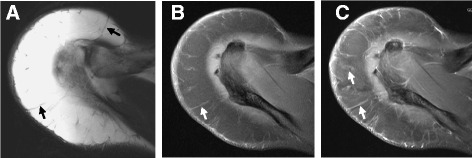
**A 40-year-old male patient with ALT measuring 15 cm in diameter.** Axial T1-weighted image **(A)** and T1- weighted fat saturation image **(B)** clearly demonstrated thick septa (arrows). T1-weighted fat saturation after gadolinium administration demonstrated enhancement of these septa **(C)**. This case obtained 5 points using the ALT score.

Adipocytic tumors represent the largest single group of mesenchymal tumors because of the high prevalence of lipomas and angiolipomas [2]. Although orthopaedic surgeons rarely encounter malignant soft tissue tumors, relatively large numbers of adipocytic tumors are found in outpatient clinics. It is well-known that adipocytic tumors, regardless of their benign or malignant status, could be large in size without inducing any symptoms. In this study, 31 out of 48 patients with lipomas had tumors larger than 5 cm. Patients with tumors less than 5 cm in size and superficially located may undergo resectional biopsy for diagnosis; however, diagnostic imaging studies are usually performed preoperatively. If computed tomography or MRI examinations reveal a soft tumor with lipomatous content in the majority of the tumor volume, lipoma or WDL/ALT is suspected rather than a high-grade soft tissue sarcoma (Figures 4 and 5). Even though the tumor may be large in size, asymptomatic lipomas do not necessarily require surgical resection. On the other hand, treatment of WDL/ALT is still controversial because of its very low malignancy potential [1,2,5,6]. Because WDL/ALTs have no potential for metastasis unless they undergo dedifferentiation, some pathologists suggest that the term “atypical lipomatous tumor” is more appropriate to use rather than “liposarcoma” [1]. The rate of dedifferentiation of WDL/ALT was previously reported to be 1–4% [6-8]. However, dedifferentiated liposarcoma (DDL) shows much more malignant potential than conventional WDL/ALT with a 5-year survival rate of 60–70% [9,10]. Okada et al. reviewed 18 cases of primary (*de novo*) DDL in the extremities and reported that the duration of the symptoms was an average of 38 months, and 9 patients showed rapid growth of long-standing tumors [10]. This result suggests that if preoperative diagnosis of WDL/ALT is easily made, surgeons could recommend resection of the tumor before it dedifferentiates.

Previously, other researchers reported on the significance of septal structures in WDL/ALT [4,11]. Gaskin et al. tried to differentiate WDL/ALTs from lipomas based upon the viewpoint that simple lipomas may contain thin, discrete septa, whereas WDL/ALTs usually contain thick or nodular septa or enhancement [12]. MRI analysis of 126 fatty masses by musculoskeletal radiologists reached the correct diagnosis in all 6 WDL/ALT cases (sensitivity, 100%); however, 10 of the suspected ALT tumor cases turned out to be variants of benign lipomas, such as chondroid lipoma, osteolipoma, or angiolipoma. The differential diagnosis of lipomatous tumors largely depended on the decisions made by the musculoskeletal radiologists. It would be useful for non-oncologist orthopedic surgeons if simplified diagnostic criteria were available. Therefore, we have created a scoring system to discriminate between lipoma and ALT by the combination of 4 values (Table 2). The score can be measured if enhanced MRI is performed (Figures 4 and 5). Based on this score, diagnosis of ALT is possible with 100% sensitivity and 77% specificity. This result is superior to MRI findings of intratumoral septa alone as a diagnostic finding, which showed 91.7% sensitivity and 74.2% specificity. Although the prevalence of hibernoma is very low, its MRI findings are similar to those of WDL/ALT. Vassos recently reported that hibernomas show spotty areas of contrast enhancement as well as prominent fibrovascular septa on MRI [13]. Hibernomas exhibit very high standard uptake values (SUVs) on [18F]fluorodeoxyglucose (FDG)-based positron emission tomography (PET) because they contain abundant mitochondria and are highly metabolically active [14]. One of our cases of hibernoma showed an SUV >40, suggesting that PET might be useful to distinguish hibernomas from WDL/ALTs (manuscript in preparation).

Treatment for WDL/ALT is still controversial because the recurrence rate after surgical resection of WDL/ALT is variable, ranging from 0–69% [15-17]. The recurrence of ALT in our series was seen in only 1 case (8.3%), similar to the findings in the report by Sommerville et al. showing an 8% local recurrence rate after marginal resection of 61 cases of ALT [6]. We agree with Sommerville et al. and Kubo et al. in the idea of “conservative” surgery for ALT to preserve the major vessels or nerves [18]. However, for recurrent ALT cases, we recommend as wide of a resection as possible because tumor margins are not usually clear and there is an increased chance of dedifferentiation. An increased number of intratumoral angiogenic vessels was revealed to be a significant factor that differentiates ALTs from lipomas in this study. Because angiolipomas are characterized by rich vasculature in mature adipose tissue, vascularity alone is not useful for the differentiation of ALTs from lipomas. As Folkmann’s group proposed, angiogenesis could be a switch that turns on the malignant phenotype in adipocytic tumors [19]. In our study, the highest number of intratumoral vessels (21.4 vessels per field) was observed in the case of ALT recurrence, which eventually dedifferentiated. Contrast enhancement MRI definitely reflects the vascular supply in the tumor and also supports the theory.

## Conclusion

Our ALT score (0–6 points) can be used to differentiate ALTs from lipomas based on MRI. If the score is equal to or higher than 3, we recommend marginal resection of the tumor to confirm the pathological diagnosis. Cut-off value should be validated by the future study because of the number of the case is not large in this study. Once the diagnosis of ALT is established, careful follow-up is recommended, especially for cases with increased vascularity.
